# Insights on Hydrogen Bond Network of Water in Phospholipid Membranes: An Infrared Study at Varying Hydration

**DOI:** 10.3390/membranes15020046

**Published:** 2025-02-04

**Authors:** Valeria Conti Nibali, Caterina Branca, Ulderico Wanderlingh, Rosaria Verduci, Elisa Bonaccorso, Andrea Ciccolo, Giovanna D’Angelo

**Affiliations:** Department of Mathematics, Computer Science, Physics and Earth Science, University of Messina, Viale Stagno D’Alcontres 31, 98166 Messina, Italy; vcontinibali@unime.it (V.C.N.); cbranca@unime.it (C.B.); uwanderlingh@unime.it (U.W.); rosaria.verduci@unime.it (R.V.); elisa.bonaccorso@studenti.unime.it (E.B.); andrea.ciccolo@studenti.unime.it (A.C.)

**Keywords:** FTIR attenuated total reflection spectroscopy, hydrogen bonding, phospholipid membrane, phase transition, membrane hydration

## Abstract

Water in membrane interphases is vital for cellular biological functions, but despite its importance, the structure and function of biological water remain elusive. Here, by studying the OH stretching mode in partially hydrated lipid multilayers by FTIR measurements, relevant information on the water structure near the surface with lipid membranes has been gathered. The water hydrogen bond network is highly perturbed in the first layers that are in contact with the lipid membrane, exhibiting strong deviations from tetrahedral symmetry and a significant number of defects, such as isolated water molecules and a large number of hydrogen-bonded water dimers in the interphase region. These findings support the hypothesis that water chains form in phospholipid membranes, and are involved in the proton transfer across lipid bilayers by phosphate groups of opposing lipids. Furthermore, we have determined that even at very low hydration levels, a small amount of water is embedded within the confined spaces of the hydrocarbon region of phospholipid bilayers, which could potentially contribute to the structural stability of the lipid membrane.

## 1. Introduction

Biological membranes are now recognized to play a major role in many cellular processes. Typically, they are composed of a mixture of different phospholipids and proteins organized in a bilayer structure, the thickness of which typically ranges from 30 Å to 50 Å, depending on lipid composition, temperature, hydration levels, and the presence of cholesterol or other modifiers [1,2].

Water is a key element that controls the self-assembly [3] and the function [4] of the lipid bilayers in the cell through complex interactions between water molecules with the hydrophilic head groups of lipids. Changes in the degree of hydration or in the organization of water molecules at the membrane interphase deeply affect the organization and dynamics of lipids in the membrane [5,6,7], the biological functions of the membrane, and the membrane-embedded proteins [8,9,10]. The vice versa is also true: for example, the simple substitution of hydrogen atoms (single protons) by methyl groups in the headgroups of the membrane lipids, as in the case of phosphatidylethanolamines and phosphatidylcholines, causes strong changes in the organization of water molecules around the amphiphiles, influencing the effectiveness of these lipids on protein activities [10].

Hydration also allows adhesion and fusion between different membranes and between membranes and proteins [11], affecting the flexibility of biomembranes [12].

Furthermore, the phase state of the membrane is highly sensitive to the water content: when it is lowered below the value corresponding to the fully hydrated condition for the lipid bilayer, the temperature of the transition from the low-temperature rigid gel phase ($L_{\beta }$) to the high-temperature (and biologically most relevant) liquid crystalline phase ($L_{\alpha }$) is shifted to higher values [13], with consequent dramatic effects on the permeability properties of the bilayer [14,15,16,17,18].

Water species are differently organized in the bilayer, and they can also be distinguished by the number and type of hydrogen bonds (HBs) coordinating water molecules among themselves and with the lipid residues [19,20].

Over the last years, experimental and simulation studies with varying water content have unveiled important details on the conformation and dynamics of lipid membranes [21,22,23]. Moreover, a slowdown of the translational and rotational dynamics of the interphase water compared to that of the bulk water has been observed [24,25]. This slowdown is even more pronounced at low hydration levels, with the lowest values for the water hydrogen bonded to the carbonyl oxygen buried deep in the hydrophobic core.

Although the structural organization and dynamics of the lipid–water system have been extensively studied for a long time [26,27,28,29,30], many of the details of membrane hydration and the water–lipid interface [31,32,33] are far from being well understood. Further insights into the interplay between membrane lipids and the aqueous environment and further elucidation of the molecular-level structure of water near the membrane/water interface are necessary to fully understand the conformational properties and stability of cell membranes and the functioning of biological cells. Studying the properties of phospholipid membranes with varying water content represents an interesting possibility to get insights into the hydration process and the formation of HBs between water and polar moieties at the interphase.

Here, we present a Fourier transform infrared-Attenuated total reflectance (FTIR-ATR) study aiming to explore the arrangement of water confined in a model lipid membrane (dimyristoyl phosphatidylcholine, DMPC), by investigating the OH stretching (ν-OH) band of water at various low-level hydration. The frequencies of this band are influenced by the HBs formed between water molecules and the polar groups of lipid headgroups, as well as among water molecules near the lipid interface. In the majority of studies, membranes are in a highly hydrated state (i.e., in the physiologically relevant, fully hydrated, or “excess water” condition), and the infrared spectra provide information on the structure of the water, averaging over the contributions from bulk and interphase.

Conversely, at low levels of water content, it is expected that the spectrum is mainly contributed to by the first hydration layers of the membrane, and water is almost fully involved in bonding to lipid groups. In such cases, the frequencies (which describe the hydrogen-bond strengths) and the structure of the ν-OH band are quite susceptible to the local environment of the water molecules at the membrane–water interface. Therefore, in the present study, the hydration-induced changes in the OH stretching band were investigated as valuable markers for the water–lipid interactions and the hydrogen bonding structure in the first hydration layers [20,34].

Our current study uses DMPC as a model system to establish a baseline hydration behavior and hydrogen bonding dynamics without the additional complexity of electrostatic interactions. This simplification allows us to focus on the intrinsic contributions of water structure and hydrogen bonding at lipid interfaces. In fact, DMPC is an electro-neutral zwitterionic lipid, containing a positively charged choline head group and negatively charged phosphate and carbonyl groups, all potentially involved in interactions with water molecules. Water molecules may also cross-link the phospholipid head groups via hydrogen bonding to the oxygens at the interphase.

Hence, the water-lipid interactions were also inspected by jointly analyzing the changes in the PO stretching of the phosphate group PO${\,}_{2}^{-}$ in the head moiety and the stretching vibrations of the polar ester carbonyl C=O groups in the interfacial region. In this way, we were able to investigate in great detail the water molecule environments of poorly hydrated membranes and the binding sites of water in biomembranes by identifying H-bonds between interfacial water molecules and phospholipid head groups [35].

## 2. Materials and Methods

DMPC was purchased from Avanti Polar Lipids (Birmingham, AL, USA) and used without further purification. A membrane-forming solution was prepared by dissolving 20 mg of dry phospholipids in 1 mL of a 2:1 chloroform/methanol solution in a flask. Then, the solution was evaporated by using a vacuum pump. After that, 1 mL of distilled water was added, and the solution was left to hydrate for about 1 h at 35 °C. Finally, it was stirred in a vortex mixer to promote the formation of liposomes.

Aligned multistacking of phospholipid bilayers was obtained by spreading about 70 μL of liposome suspension directly onto the ATR plate and evaporating water. The sample was repeatedly subjected to hydration/dehydration cycles. Hydration introduces water molecules into the lipid layers, encouraging the formation of stable bilayer structures, while dehydration allows for the controlled collapse and reorganization of these layers. In fact, while evaporating the aqueous solvent, capillary forces flatten the membranes, which spontaneously form highly aligned multibilayers [36].

It is worth noting that, while multistacked phospholipid membranes do not fully replicate the curvature and dynamics of cellular membranes, they effectively model hydration and interfacial processes occurring in stacked lipid layers, such as those found in lamellar bodies or other densely packed lipid environments.

Saturated salt solutions, prepared by mixing a great excess of chemically pure salt and deionized water, were used to maintain the sample at different relative humidity (RH). For the experiments, the solution was poured into a small toroidal tank set around the ATR plate, and the whole area was isolated from the rest by a sealed cover.

To ensure that the membrane was in a well-equilibrated hydrated state, it was kept at room temperature for 24 h or more at each hydration before measurements until the absorbance spectrum stabilized in the region 2800–3800 cm${\,}^{-1}$. Furthermore, for each salt solution, hydration and dehydration scans were acquired, and negligible hysteresis effects were observed. All measurements were carried out in duplicates by preparing new lipid solutions each time.

For this study, nine salts were used (LiCl, CH${\,}_{3}$COOK, MgCl${\,}_{2}$, K${\,}_{2}$CO${\,}_{3}$, NH${\,}_{4}$NO${\,}_{3}$, NaCl, KCl, KNO${\,}_{3}$, K${\,}_{2}$SO${\,}_{4}$) to have a range of RH ranging from 11 to 98%. The values of equilibrium RH at 25 °C are given in Table 1.

The molar water-to-lipid ratio $n_{w}$ has been determined by gravimetric sorption experiments [37] at a temperature of T = 25 °C. The adsorption isotherms of DMPC at various hydration levels were measured using an electrobalance operating in a remote weighing configuration with its weighing mechanism separated from the control unit. Approximately 200 mg of DMPC was placed in the balance, which was enclosed in a sealed chamber. The chamber, maintained at 30 °C, allowed control of RH using saturated salt solutions to establish specific hydration levels. Weight measurements were recorded remotely as the samples equilibrated at each humidity condition. Phospholipid samples were equilibrated over saturated salt solutions at specific RHs for 48–72 h. The equilibrated samples were weighted to obtain the initial weight $W_{wet}$, then dried at 60–70 °C in a vacuum oven for 24–48 h and reweighed to determine the dried weight $W_{dry}$. The water content formula $W_{water}=W_{wet}-W_{dry}$, and the percentage of water content $\%W_{water}=\left(W_{water}/W_{wet}\right)\times 100$ were calculated. The molar ratio of water to phospholipid was determined from the ratio of water moles to lipid moles based on their molecular weights. Finally, the number of water molecules per lipid $n_{w}$ was computed by the equation

(1) $$n_{w}=\frac{n_{water}}{n_{lipid}}=\frac{\left(\frac{W_{water}}{M_{water}}\right)}{\left(\frac{W_{dry}}{M_{lipid}}\right)}$$

and simplifying

(2) $$n_{w}=\frac{\left(W_{water}\cdot M_{lipid}\right)}{\left(W_{dry}\cdot M_{water}\right)}$$

This method was adapted from the experimental procedure described by Jendrasiak and Hasty [37]. Measurements were repeated in triplicate for reproducibility.

The values obtained for $n_{w}$ were reported in Table 1. They were found to range from 1.25 (lowest hydration) to 8.5 (highest hydration), in good agreement with previous results [38,39].

**Table 1:** Selected saturated salt solutions and corresponding RH at 25 °C along with the respective number of water molecules per DMPC evaluated by gravimetric sorption experiments, $n_{w}$, and infrared area under the bands corresponding to the CH${\,}_{2}$ stretching vibration, $n_{w}^{IR}$. Note that the observed good agreement between $n_{w}$ and $n_{w}^{IR}$ suggests that water molecules enter uniformly between the lipid bilayers preserving both the lamellar lipid structure and the good bilayer alignment. If it were not so, there would be defects in the alignment of lipids, that should result in the formation of cavities where water molecules can accumulate at the expense of lipids [40]. The presence of defect-induced pools of water molecules would cause a more intense absorption signal for the water band, and a consequent less intense absorption intensity for the methylene band, preventing the IR-derived data from being in good agreement with the gravimetric data.

Salt	%RH	$n_{w}$	$n_{w}^{IR}$
LiCl	11	1.25	1.2
CH${\,}_{3}$COOK	23	1.70	1.73
MgCl${\,}_{2}$	33	2.10	2.1
K${\,}_{2}$CO${\,}_{3}$	43	2.40	2.78
NH${\,}_{4}$NO${\,}_{3}$	64	3.33	3.41
NaCl	75	4.00	3.89
KCl	85	4.97	4.76
KNO${\,}_{3}$	95	7.00	7.52
K${\,}_{2}$SO${\,}_{4}$	98	8.51	8.51

DMPC bilayer has a gel-to-liquid crystalline transition temperature of 24 °C in its fully hydrated state (25–30 water molecules per lipid). The phase state is highly susceptible to the content of water (lyotropic phase behavior); when the water/lipid molecule ratio is lower than twelve, the order among the hydrocarbon chains increases and the main transition temperature is moved toward higher values so that partially hydrated multilayers are in gel phase at temperatures at which they would be in liquid crystalline phase. For the conditions at which the studies were performed (T = 25 °C and $n_{w}$ at most equal to 8.5) lipid bilayers are considered to be in a gel phase.

## 3. Experimental Section

Fourier-transform infrared spectra were taken at 25 °C in attenuated total reflectance (ATR) mode using a single reflection horizontal ATR accessory, having a diamond crystal fixed at an incident angle of 45° (Platinum ATR, Bruker, Billerica, MA, USA) mounted on a Vertex 80V FT-IR spectrometer (Bruker Vertex 80V). The penetration depth (*d*${\,}_{p}$) of IR light in the sample is the following:

(3) $$d_{p}=\frac{\lambda }{\left(2\pi n_{1}\sqrt{\sin^{2}\left(\theta \right)-{\left(n_{2}/n_{1}\right)}^{2}}\right)}$$

where λ is the wavelength of the incident light, *n*${\,}_{1}$ is the real refractive index of the internal reflection element (*n*${\,}_{1}$ = 2.4), θ is the angle of incidence and *n*${\,}_{2}$ is the refractive index of the sample. The latter parameter was evaluated by the formula

(4) $$n_{2}=\frac{1.33V_{W}+1.44V_{L}}{V_{W}+V_{L}}$$

where $V_{W}$ and $V_{L}$ are the molar volumes of water and lipid, respectively, while 1.33 and 1.44 are the corresponding refractive indexes. Consequently, *n*${\,}_{2}$ varies, switching from the dried sample (*n*${\,}_{2}$ = 1.44) to the most hydrated sample (*n*${\,}_{2}$ = 1.41), which only slightly affects *d*${\,}_{p}$, that, for all the different hydrations, it was estimated to be around 400 nm for a wavelength of 3.0 μm, close to the peak of the OH stretching band.

Based on the above considerations, the thickness of the stack of lipid bilayers, of about 10 μm, exceeds several times the value of *d*${\,}_{p}$ of the evanescent wave at all the investigated hydrations.

A background scan was recorded before each measurement and subtracted from the sample spectra. Each spectrum was averaged over 216 scans with a resolution of 2 cm${\,}^{-1}$ and ATR corrected. Atmospheric moisture effects were eliminated by performing measurements in an evacuated optics bench configuration.

The values of the number of water molecules per lipid $n_{w}^{IR}$ at each hydration were determined from infrared spectra by evaluating the ratio of the total area of the methylene band in hydrated samples, $A_{CH,wet}$, to the total area of this band in the dry sample, $A_{CH,dry}$, and the fraction of volumes available to water molecules: $n_{w}^{IR}$ = (1 − ($A_{CH,wet}/A_{CH,dry}$)$V_{L}/V_{W}$). As shown in Table 1, the values of $n_{w}^{IR}$ obtained by this procedure are in agreement within a few percent with those determined experimentally by gravimetric sorption measurements.

The present infrared analysis investigated the water-lipid H-bonding interactions and hydration processes from two different perspectives: the water and the lipid one. From the water perspective, the focus was on the shape and on the frequency shifts of the hydroxyl (OH) stretching band at varying hydration levels, which are related to the number and strength of hydrogen bonding of different water populations in the hydration sites of the membrane, with the highest frequency due to the weakest interactions of water molecules.

Conversely, the changes of the stretching vibrations of the polar ester carbonyl C=O groups in the interfacial region and the phosphate group PO${\,}_{2}^{-}$ in the head moiety, were monitored from the lipid perspective [7,41]. Both of these groups are sites of water sorption and are proton-acceptors: when water molecules bond with them through hydrogen bonding, their stretching frequencies decrease markedly due to the elongation of the involved chemical bonds [42].

## 4. Results and Discussion

**Figure 1:**
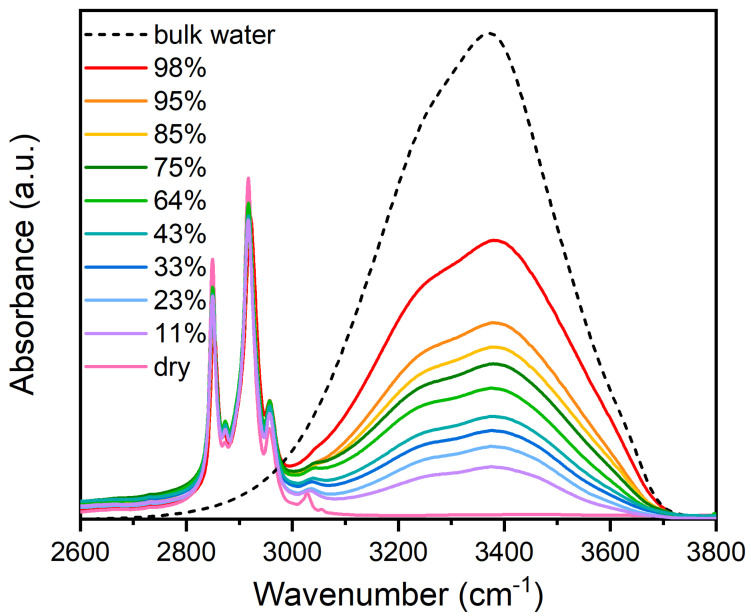
Comparison of the OH stretching band shape in the extended spectral range of 2600−3800 cm${\,}^{-1}$ for pure liquid water (H${\,}_{2}$O) and for DMPC multibilayers hydrated at different RH values. The spectra are normalized to the CH${\,}_{2}$ /CH${\,}_{3}$ stretching band (2800−2970 cm${\,}^{-1}$).

Figure 1 shows the spectral region between 2600 and 3800 cm${\,}^{-1}$ of DMPC bilayers at different hydration levels, from dry to 98%, compared to the spectrum of pure liquid water. In this region, pure water shows the characteristic broad band vibrational spectrum due to the OH stretching vibrations with a maximum near 3400 cm${\,}^{-1}$ and a low-frequency wing near 3200 cm${\,}^{-1}$ [43].

In the hydrated lipid sample, besides the OH stretching band (ν-OH), the typical methylene stretching (CH${\,}_{2}$) bands of acyl chains are also revealed below 3000 cm${\,}^{-1}$ [20,34]. To account for the swelling behavior, all the spectra were normalized to the CH${\,}_{2}$ /CH${\,}_{3}$ stretching band (2800–2970 cm${\,}^{-1}$). In fact, in an ATR experiment, it is common to observe an increase in the OH stretching band and a decrease in the C-H bands when the hydration level increases [44]. This rise and fall of the band magnitudes can be accounted for by considering that the gradual water absorption induces the progressive swelling of multibilayers. Consequently, a major quantity of water molecules and a minor content of lipids are present in the sampled volume.

It is clear from Figure 1 that the shape of the ν-OH band observed in the spectral region above 3000 cm${\,}^{-1}$ in hydrated lipids differs from that in pure water and depends on the relative hydration level.

**Figure 2:**
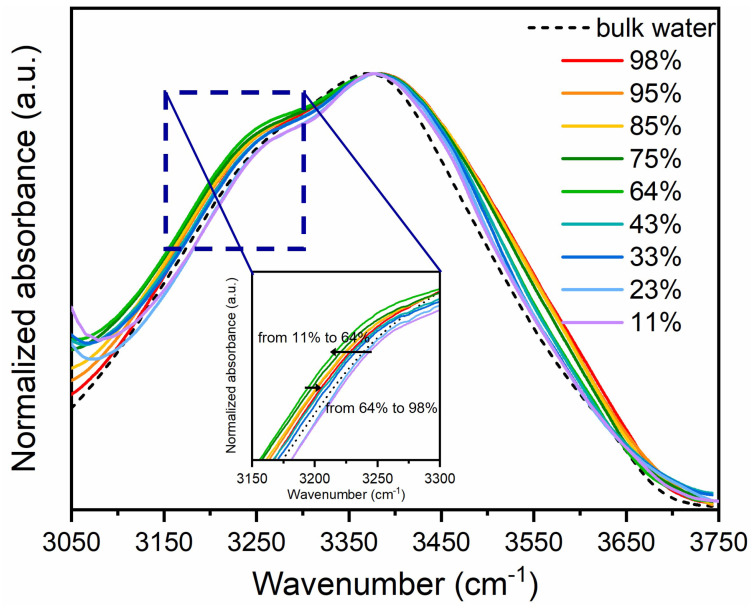
Comparison of the OH stretching band profile for pure liquid water and DMPC at different RHs, after normalization to the maximum amplitude.

To better appreciate the changes in the ν-OH band at different RHs, the experimental data were normalized to the maximum amplitude and plotted in Figure 2.

The comparison with the spectrum of pure liquid H${\,}_{2}$O shows that the shape of the OH-stretching band strongly differs in both the high- and the low-frequency sides relative to the peak at 3420 cm${\,}^{-1}$ in pure water. Furthermore, the changes are dependent on the water molecules content. These observations indicate that the water hydrogen-bonded structure is strongly changed in the lipid system with respect to the pure liquid water as a consequence of the interaction with the hydrophilic-hydrophobic groups [3]. In particular, as the water content increases, in the membrane system the high-frequency shoulder near 3600 cm${\,}^{-1}$ becomes gradually more evident, appearing to shift to higher frequencies and showing a major increase at RH = 75%. Differently, the intensity of the low-frequency component, centered at about 3250 cm${\,}^{-1}$, progressively increases as RH increases up to 64%, whereas it reverses the trend as RH is increased above 64% (see the inset of Figure 2). The redshift in the OH stretching band at low hydration indicates the dominance of tightly bound water molecules forming strong HBs with lipid headgroups. Conversely, the blueshift observed at high hydration corresponds to a transition toward bulk-like water, characterized by weaker HBs and extended hydrogen-bonding networks.

Furthermore, the position of the main peak at about 3420 cm${\,}^{-1}$ is shifted to higher frequencies with respect to that of liquid water. This finding would indicate the weakening of the average H-bonding in lipid bilayers.

Arsov et al. [45,46] proposed that weakened HBs in confined water could contribute to a long-range attractive hydration force, which competes with van der Waals and fluctuation forces at equilibrium interlamellar spacing. This force arises from the restructuring of water and the resulting negative work required to transfer confined water into the bulk phase. Our results support the notion of a perturbed water structure near the interface, which could extend the range of hydration forces. This restructuring likely influences not only hydration forces but also the steric and fluctuation forces that govern bilayer interactions.

To provide a quantitative analysis of these changes to obtain information on the various types of OH species present in water, as well as to clarify intermolecular interactions and the influence of micro-confinement, a deconvolution procedure of the ν-OH band was conducted.

Differently from previous works [47,48], here, the band was decomposed into five contributions by considering that the molecules involved in the formation of HBs act as either proton donors (D) or proton acceptors (A). This approach aligns with well-established precedents in the literature, which identify hydrogen-bonding configurations such as free OH, weakly and strongly hydrogen-bonded water molecules, water dimers, and tetrahedral structures. Previous studies using Raman spectroscopy consistently support this methodology [49,50]. By distinguishing these five populations, we aim to capture the structural heterogeneity of water in the interphase region, providing deeper insights into hydration mechanisms. The five components, labelled as DAA, DDDA, DA, DDA, and free OH, correspond to water molecules having different H-bond coordination numbers (ranging from 0 to 4) according to the number of H-bond donor (D) sites and H-bond acceptor (A) sites [51,52,53]. For example, in a DDAA configuration, a molecule can simultaneously donate and accept two HBs. The structure of the DA bond patterns is linear, whereas the other molecular models, DDAA, DDA, and DAA, create three-dimensional networks of HBs. Also, water molecules with stronger HBs and higher connectivity give rise to lower frequency O–H stretching sub-bands, while water with weaker HBs and lower connectivity result in higher frequency sub-bands [54,55].

For the deconvolution of the ν-OH band in hydrated lipids, an additional low-frequency Gaussian component was included to account for the contributions of the high-energy tail of CH${\,}_{2}$ groups and the asymmetric stretching mode of the N(CH${\,}_{3}$)${\,}_{3}$ head groups observed below 3050 cm${\,}^{-1}$ [56].

**Figure 3:**
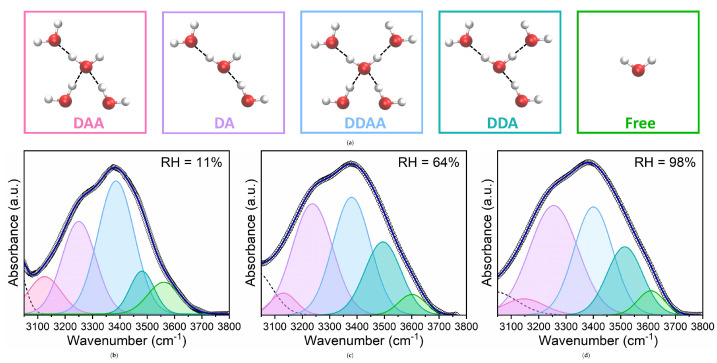
(**a**) Schematic representation of the different hydrogen bonding structures of water and the free water molecule; and (**b**–**d**) Gaussian deconvolution of the OH stretching band of DMPC/H${\,}_{2}$O sample at three selected hydration levels.

Figure 3 shows an example of the OH band decomposition into five Gaussian sub-peaks for the DMPC at three selected hydration levels (low, intermediate, and high). Fitting has provided unique solutions independently of the starting values of parameters. Moreover, the high quality of fit obtained for our experimental spectra further validates the adoption of this approach. Our FTIR studies reveal that the ordering and arrangement of the HBs in the water layers adjacent to the phospholipid head groups are significantly influenced by lipid hydration and exhibit clear heterogeneity.

**Figure 4:**
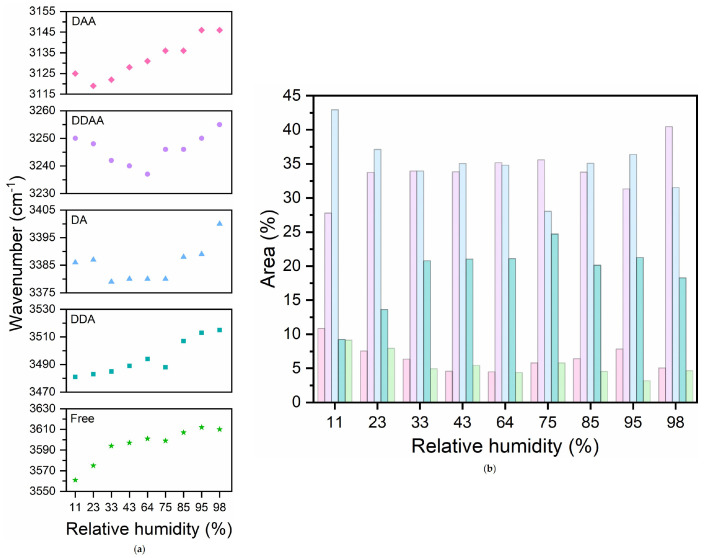
(**a**) Frequencies and (**b**) relative areas of the five OH components obtained by fitting Gaussians to the experimental OH stretching band at the investigated hydration levels. DAA, DDAA, DA, DDA and Free OH components are colored in pink, light violet, light blue, dark cyan and light green, respectively.

From the fitting procedure, we have estimated the relative contribution of each Gaussian area to the total area of the ν-OH band (percentage areas) together with the peak frequencies of sub-bands. The results are shown in Figure 4.

We observed that the DDAA and DA bands are the most prominent at all investigated hydrations, contributing to more than 70% of the total integrated area of the ν-OH band. Notably, although DA dimer populations prevail, a considerable number of water molecules are arranged in tetrahedral DDAA substructures even at very low hydration levels. Surprisingly, at these low hydrations, a non-negligible significant number of free waters is also found.

Furthermore, in lipid bilayers the position of the DA is roughly unchanged at around 3380 cm${\,}^{-1}$ up to RH = 75% and then increases to 3400 cm${\,}^{-1}$. Conversely, the position of the low-energy DDAA component exhibits a redshift (from 3250 to 3235 cm${\,}^{-1}$) as RH increases up to 75%, and a blue shift (up to 3258 cm${\,}^{-1}$) for RH > 75%, whereas the positions of the other components remain fairly unchanged or increase slightly.

Considering that the linear absorbance is proportional to the number density of resonant oscillators, to better describe the obtained results, we have calculated the number of water molecules ($n_{w}^{\prime }$), associated with each of the five OH environments, by multiplying the percentage areas for the number of water molecules reported in Table 1 and corresponding to the different RHs.

In this regard it is worth noting that, to correctly evaluate the intensity of infrared absorption of OH stretching vibrations, it should be necessary to quantify the variation of the OH transition dipole as a function of the OH frequency. In confined water molecules, the dipole moment magnitude increases with the number of HBs they form, varying at most from a value of about 2.9 D (for molecules that self-organize through HBs) to a value of 2 D (for isolated water molecules in small cages) [57].

Nevertheless, while these values should be regarded as approximate due to the unknown dipole moments for the different vibrations of water confined within lipid bilayer, the water population distributions we have determined provide valuable insights into how hydration occurs within the different hydrogen bonding environments near the membrane interface.

**Figure 5:**
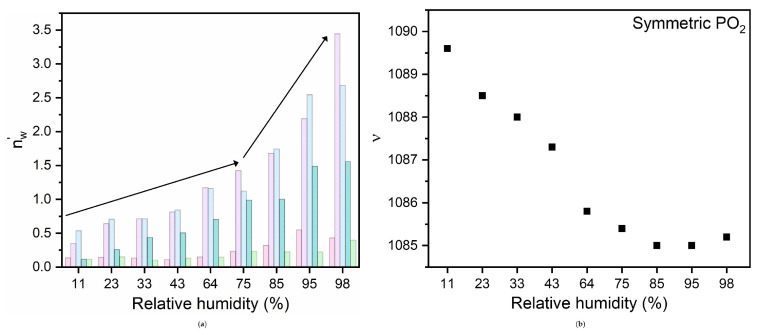
(**a**) Number of water molecules, ($n_{w}^{\prime }$), associated with the five water environments revealed by the fitting routine as a function of hydration state (the data are reported as histograms for easier visualization). DAA, DDAA, DA, DDA and Free components are pink, light violet, light blue, dark cyan and light green, respectively; (**b**) hydration dependence of the frequency of the PO${\,}_{2}^{-}$ symmetric stretching band of hydrated DMPC.

Indeed, the results shown in Figure 5a disclose important information. First, despite its percentage of surface area tending to decrease (Figure 4b), the number of free water molecules increases monotonically with increasing hydration (light green bars Figure 5a). More importantly, the numbers of water molecules associated with the DDAA and DA components both increase, exhibiting a greater slope when the hydration level overcomes the value of 75% (Light violet and light blue bars, Figure 5a).

As is well known, water molecules in the lipid bilayer can occupy different regions, resulting in varying degrees of binding strength: some amount of water resides deep within the lipid-water interface by binding strongly to PO${\,}_{2}$, and more weakly to CO [58]; a limited number of water molecules is found confined between the hydrocarbon chains of the bilayer (buried water); water molecules are also found near the N(CH${\,}_{3}{)}^{3+}$ groups and are involved in a clathrate-like structure [15,16,17,18].

To correlate the H-bonding formation at PO${\,}_{2}^{-}$ with the observed changes in the sub-bands, we have carefully analyzed the stretching vibrations of the phosphate group representing the primary hydration site of PC lipids (lipid perspective) [34].

For such analysis we have considered the symmetric PO${\,}_{2}^{-}$ stretching band, because in contrast with what was observed for the asymmetric band [7], it is well-resolved at all the hydrations. The evolution with hydration of this band is shown in Figure 5b. As expected [7,41], the frequency position gradually decreases, indicating the progressive water uptake at the PO group and the related formation of HBs, and becomes almost constant in the range of RH = 75%, suggesting that, beyond this hydration level, the formation of HBs for this group is saturated.

The observed similar decreasing trend of the νPO${\,}_{2}^{-}$ and the frequency position of the DDAA band with varying RH values indicates that the related molecular events are strictly correlated and/or are the two different faces (of the water and of the lipid ones) of the same mechanism and suggests that the formation of HBs at PO${\,}_{2}^{-}$ sites is involved in the four-coordinated tetrahedral water environments.

**Figure 6:**
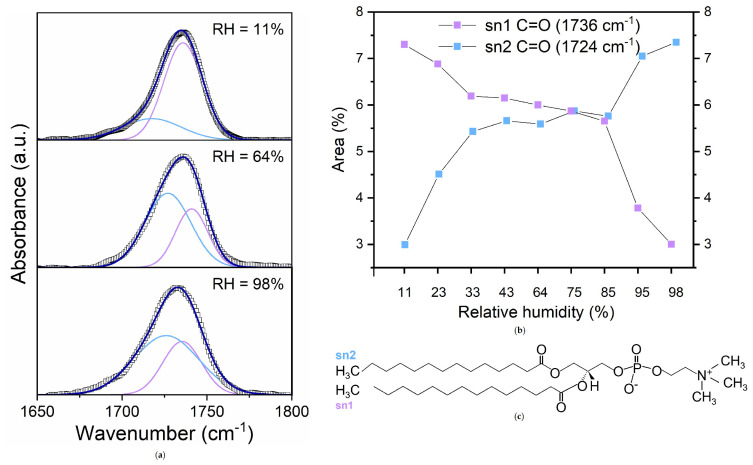
(**a**) Deconvoluted infrared absorbance spectrum of DMPC in the 1650−1800 cm${\,}^{-1}$ region at three selected hydration levels; (**b**) Area percentages of the hydrogen and non-hydrogen bonded C=O groups and (**c**) structure of DMPC molecule. sn-1 C=O and sn-2 C=O groups are colored in light violet and light blue, respectively.

To further inspect the hydration sites of lipids at the interface, the spectral region of the stretching band of the ester carbonyl group, extending between 1800 and 1650 cm${\,}^{-1}$ has been also scrutinized (Figure 6).

In this region, at all the investigated hydrations, an asymmetric band is observed that broadens and shifts towards lower wavenumber with increasing the water content. Indeed, this band comes from the overlapping of a band at 1741 cm${\,}^{-1}$ and a band at 1727 cm${\,}^{-1}$, corresponding to the C=O stretching vibrations of the ester groups at the sn-1 and sn-2 positions and, respectively, describing non-hydrogen-bonded and hydrogen-bonded carbonyl group [13,41,59].

In Figure 6a, the stretching band of the carbonyl group, deconvoluted into two Gaussian contributions from hydrogen and non-hydrogen-bonded C=O groups at three selected hydrations, is shown. The absorbance values of the two peaks were converted to percentage areas to obtain the percentages of hydrogen-bonded and non-hydrogen-bonded C=O groups (Figure 6b).

It is found that the area of sn-2 C=O groups increases with increasing RH, remains about constant between 40 and 85%, and then starts to grow again. A mirrored trend is found for the sn-1 C=O component. It is worth noting that the abrupt increase of the sn-2 C=O area is found in correspondence with the observed saturation of hydrogen bonding sites in the PO moiety.

Interestingly, precisely in proximity to RH = 85%, the frequency at which the DDAA sub-band is observed shows a reverse trend with RH. The shift to higher frequencies could be interpreted as an overall weakening of the H-bonding among water molecules, indicating the formation of weaker HBs with C=O groups.

Our study aligns well with the previous study of Vancuylenberg et al. [60], proposing a three-water-layer model for lipid membranes: (1) headgroup-near water, (2) perturbed water, and (3) free water. In fact, our data reflect three distinct hydration behaviors:

- Low RH (<64%): Dominance of headgroup-near water, evident in strong hydrogen bonding with lipid headgroups, indicated by the high proportion of hydrogen-bonded C=O groups (Figure 6a).
- Intermediate RH (64%): Transition to perturbed water, marked by a redistribution of hydrogen-bonded and non-hydrogen-bonded C=O groups and increased spectral heterogeneity (Figure 5a). This suggests a gradual shift in water interaction dynamics.
- High RH (>64%): Stabilization in hydrogen-bonding populations and the emergence of free water reflected by the formation of a bulk-like water layer beyond the perturbed region.

These findings emphasize the dynamic interplay between water layers and their implications for membrane mechanics. At low hydration, the prevalence of headgroup-near water enhances bilayer stiffness, stabilizing its gel phase. In the transition zone, perturbed water increases flexibility, facilitating phase changes. Finally, free water at high hydration enhances fluidity and reduces intermembrane interactions. By connecting our observations to Vancuylenberg’s model, we contribute to a deeper understanding of the membrane/water nanostructure and its influence on membrane mechanics.

Notably, we also know that the gel phase of DMPC can absorb up to 13 water molecules per DMPC [11,40,61], four of which lie in the interfacial water region and are shared by two adjacent lipids: two are located between phosphate oxygens of two neighboring lipid molecules, forming an infinite hydrogen-bonded phosphate-water-phosphate-water ribbon, whereas the other two molecules are hydrogen-bonded to one phosphate oxygen of a molecule and to another water molecule, respectively, establishing a linkage across the bilayer interface with the corresponding water molecules of the adjacent bilayer [62].

From our results, the value of RH = 85% corresponds to a number of about five water molecules for lipid (see Table 1), mainly equally distributed in DDAA and DA populations.

Hence, we deduce that during the early stages of the hydration process, most water molecules are involved in interactions with the phosphate group in DDAA and DA arrangements, even if some loosely bonded water molecules are in the buried region between the hydrocarbon tails and at the carbonyl sites. A second step of hydration is activated after the saturation of phosphate groups, with the formation of a great number of HBs with the carbonyl group.

A relevant result of our analysis was the occurrence in the first layers of hydration of a high fraction of dimer water molecules, which can result in the formation of segmented water chains (water wires) with strong intradimer and weak interdimer HBs. This finding supports the hypothesis that water chains are formed in phospholipid membranes and are involved in the proton transfer across lipid bilayers by phosphate groups of opposing lipids [63,64]. Interestingly, very recently it has been shown that the presence of water dimers in the interfacial structure of water has a direct impact on the rate of water splitting [53,65]. All these findings highlight the importance of loosely constrained water structures in ion transport mechanisms under atomic confinement.

Our study also shows that at very low hydration levels, water molecules are present in the buried region between the hydrocarbon tails. If we consider that DMPC at a low hydration state and room temperature is in a gel phase, this result is in contrast with that found by Alarcon et al. [19], who estimated a negligible contribution of buried water in the gel phase of Dipalmitoylphosphatidylcholine (DPPC) bilayers by means of molecular dynamics simulations.

Significantly, a recent simulation study on the hydration of DMPC has revealed different binding states of water molecules and found that, at low hydration, most of the water is maintained inside the membrane, assuring the structural stability of the bilayer [25]. Water molecules residing in internal cavities located in the apolar hydrocarbon region are becoming recognized increasingly as having an important role in regulating the membrane fluidity and the permeability of hydrophilic non-electrolytes [66].

Experimentally, the presence of water within the hydrocarbon region of phospholipid bilayers in the fully hydrated liquid-crystalline phase membranes has been well established [67,68,69,70], but there is limited and non-definitive evidence for this in partially hydrated or gel-phase membrane [19]. The present study fills this gap and focuses on the relevance of sub-constrained water molecules at the lipid–membrane interface.

A deeper understanding of water-lipids interactions in partially hydrated membrane could enable better comprehension of the consequences of biological membrane dehydration induced by freezing or by interaction with other biomolecules, i.e., cholesterol, peptide, protein, or drug [71], or by sugar or salt, or metal ion.

Furthermore, it can shed light on the mechanisms by which some organisms, including tardigrades, survive in a dehydrated environment for long times [72] and on the fundamentals of long-time biopreservation [73]. In addition, studying the dehydration of lipids at the membrane interface can also be useful to understand the mechanism of raft domain formation [74].

## 5. Conclusions

The H-bonding characteristics of the hydration water of DMPC were studied using infrared spectroscopy by varying the RH of the ambient atmosphere of multibilayer samples. Relevant information on the hydration interaction of phospholipid bilayers has been gathered. Deconvolution of the OH stretching band in poorly hydrated lipid multilayers has allowed us to distinguish five water molecule populations characterized by different combinations of hydrogen bond donors and acceptors, providing a more detailed picture of water structuring at the membrane interphase.

We have deduced that also at very low hydration, a small amount of water is embedded in the confined spaces within the hydrocarbon region of phospholipid bilayers. This finding provides support to the results of a recent simulation study [25] and suggests that the presence of water in the interphase region and, even if in a small amount, between the hydrocarbon region determines the structural stability of the lipid membrane.

According to the study, there is a substantial amount of dimer water molecules present in the first layer of hydration, which can lead to the development of hydrogen-bonded water-wires.

The hydration process was also investigated by observing the changes of the phosphate group symmetric stretching and C=O stretching modes, correlating them with shifts in the OH stretching sub-bands and the distribution of water populations.

Our study builds upon and complements the work by Arsov et al. [45], which investigates a broad range of hydration levels. While Arsov et al. [45] focus on both excess water and partially hydrated states, our work narrows the scope to low hydration conditions, staying below the full hydration threshold. This focus allows us to provide detailed insights into tightly bound water and its contributions to hydration forces. Furthermore, we extend the spectral analysis of the OH stretching band through normalization and deconvolution, offering a more comprehensive view of water restructuring. These approaches refine our understanding of hydrogen bonding and lipid–water interactions in low-hydration regimes, providing a valuable contribution to the field.

The results of this study can be useful for understanding how changes in the degree of hydration can threaten the stability and biological function of lipid membranes and clarify the aspects of water H-bonding self-assembly and their role in transport mechanisms across lipid membranes.

## Data Availability

The data presented in this study are available upon request from the corresponding author.

## Abbreviations

The following abbreviations are used in this manuscript:

FTIR-ATR	Fourier transform infrared-Attenuated total reflectance
DMPC	Dimyristoyl Phosphatidylcholine
RH	Relative Humidity
HBs	Hydrogen Bonds
